# Approaches to developing and implementing a molecular diagnostics stewardship program for infectious diseases: an ASM Laboratory Practices Subcommittee report

**DOI:** 10.1128/jcm.00941-24

**Published:** 2024-10-21

**Authors:** Frances Valencia-Shelton, Neil Anderson, Elizabeth L. Palavecino, Maria E. Navas, Paige M. K. Larkin, Rosemary She, Laura M. Filkins

**Affiliations:** 1 Baptist Health Medical Center, Jacksonville, Florida, USA; 2 Department of Pathology, University Hospitals Cleveland Medical Center, Cleveland, Ohio, USA; 3 Department of Pathology, Wake Forest University School of Medicine, Winston-Salem, North Carolina, USA; 4 VA Northeast Ohio Healthcare System, Cleveland, Ohio, USA; 5 American Society for Microbiology, Washington, DC, USA; 6 Department of Pathology, City of Hope, Duarte, California, USA; 7 Department of Pathology, University of Texas Southwestern Medical Center, Dallas, Texas, USA; Vanderbilt University Medical Center, Nashville, Tennessee, USA

**Keywords:** diagnostic stewardship, molecular microbiology, test utilization

## Abstract

Diagnostic stewardship (DxS) for infectious disease testing requires a multi-disciplinary approach to optimize test selection, performance, interpretation and patient treatment. Nucleic acid amplification-based tests for the diagnosis of infectious diseases, or “molecular microbiology tests,” have rapidly expanded over the past two decades. With the increased availability and complexity of these tests, there is also an increased need for collaborative approaches to optimize test use to promote positive impacts on patient care, while mitigating potential negative impact or resource waste. In this review, we provide recommendations on building collaborative DxS teams, including microbiologists and the diverse stakeholders that use and interpret molecular microbiology tests. We then detail approaches to identify high-priority molecular microbiology tests that may need utilization assessment, select appropriate diagnostic stewardship interventions, and monitor the impact of implemented interventions. This strategic process may be employed by laboratories to realize optimal testing for selected tests at their institution.

## INTRODUCTION

Diagnostic stewardship (DxS) is a process employed to optimize clinical laboratory testing, thereby improving patient care. Clinical laboratory testing requires optimization during the pre-analytic, analytic, and post-analytic phases. Therefore, DxS has a role in each phase: test selection/orders, specimen collection, laboratory analyses, result reporting, and clinical interpretation and management (1).

All phases of testing are most successful with frequent review of processes and collaboration with clinical teams and other stakeholders to ensure their clinical utility (2). Most processes during the pre-analytic phase of testing are performed by non-laboratory caregivers. Therefore, consistent communication and collaboration with these teams should be a key focus of stewardship efforts. Molecular testing, i.e., nucleic acid-based testing, for the diagnosis of infectious diseases (referred to here as molecular microbiology tests) is a high priority for DxS for many reasons that include rapid change, increasingly broad availability at the point of care, high costs, and frequent lack of consensus guidelines on when and who to test. These features make molecular microbiology tests ripe for overuse in settings with low pre-test probability or where the result is unlikely to change clinical management. For example, reports demonstrate that in the absence of DxS, molecular respiratory panel testing in the emergency department (ED) does not significantly affect antiviral use, length of stay, patient disposition (3), or antibiotic utilization (4). Other examples of molecular microbiology tests with poor clinical utility in the absence of DxS include 16S rRNA PCR with sequencing (with reported clinical impact in 5% of cases) and cell-free DNA metagenomic next-generation sequencing from plasma (with reported positive impact in about 7%–12% of patients and negative impact in about 4%–5%) (5–7). DxS is needed to promote optimal, high-yield use of such molecular tests while discouraging non-clinically impactful, low-yield test utilization. As will be discussed, diligent efforts in DxS can greatly benefit patients, caregivers, laboratories, institutions, and payors alike.

**Fig 1:**
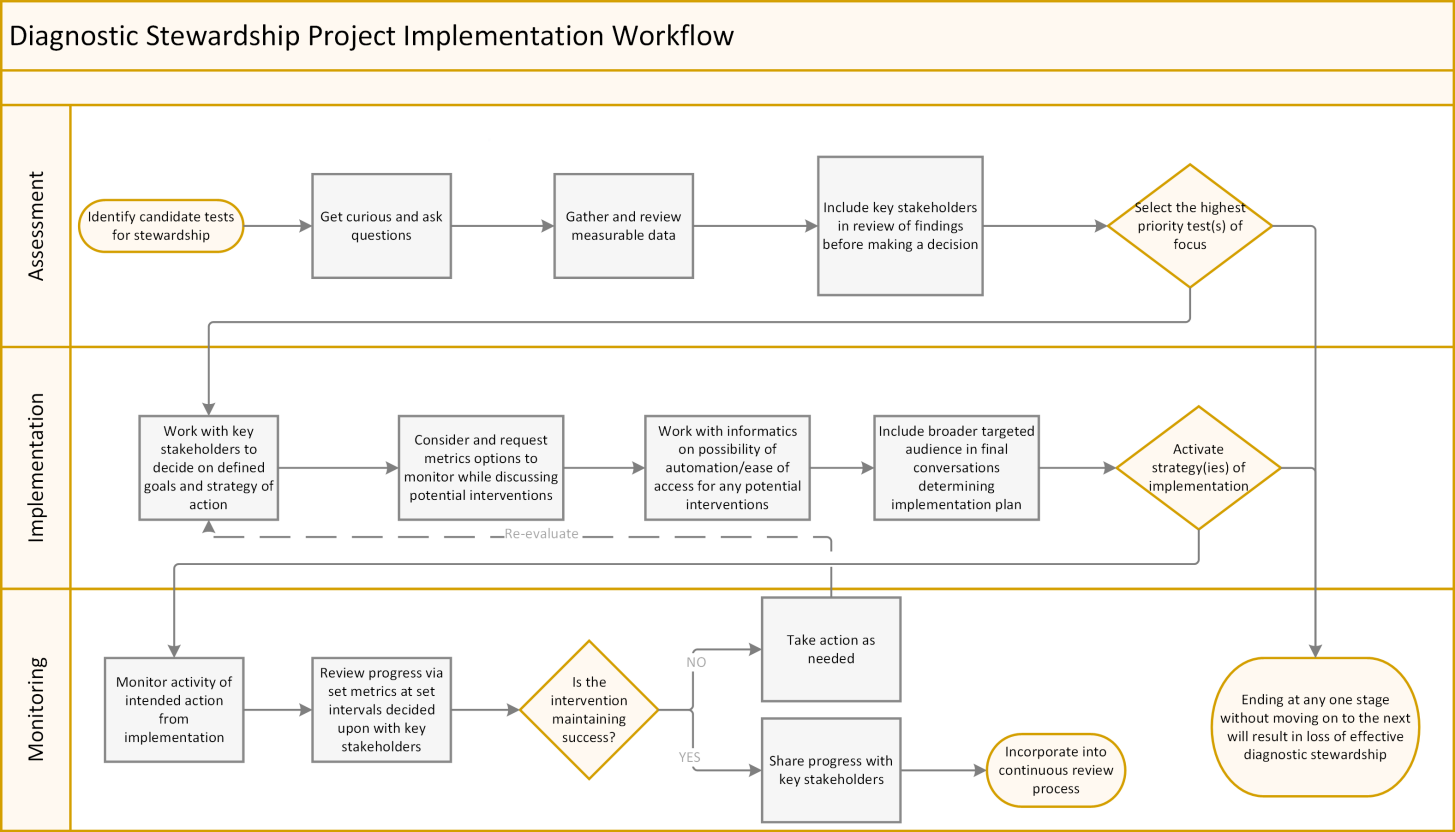
Stepwise process toward diagnostic stewardship. Effective DxS includes assessment, implementation, and monitoring phases. Assessment includes first identifying candidate tests and then using institutional data and input to prioritize efforts. Interventions should be selected to target the identified utilization practices to be modified and that can realistically be implemented with available resources. After go-live of any intervention, the impact of the intervention should be monitored. Monitoring should include metrics to evaluate for both intended and unintended consequences.

At many institutions, DxS depends upon a pathology-wide committee that handles all laboratory testing, including microbiology testing (8). As such, clinical microbiology professionals are well positioned to lead DxS efforts for infectious disease molecular diagnostic tests due to their laboratory and clinical expertise, existing partnerships with stakeholders, access to institutional utilization data, and knowledge of the financial impacts of testing. There are numerous approaches to developing, implementing, and monitoring DxS for molecular microbiology tests, but DxS can also be resource intensive. Institutions must prioritize tests for DxS and select approaches that maximize available resources. To aid clinical microbiology professionals through this process, we review DxS models that have been used and recommend step-wise methods to identify high-priority tests that may benefit from DxS. Furthermore, we describe approaches to implementing DxS practices and mechanisms to monitor the success, need for modification, or unintended consequences of DxS for molecular microbiology tests (Fig. 1).

## PARTNERSHIP WITH INFECTIOUS DISEASES PROVIDERS

For the hospital-affiliated microbiology laboratory, partnership with infectious diseases (ID) specialists is essential to broaden clinician-facing support for stewarding utilization of molecular microbiology assays. ID experts often share similar goals with clinical microbiologists regarding proper test utilization, interpretation of results, and consequent antimicrobial stewardship (AS). For example, the detection of microorganism DNA or RNA in a patient specimen does not necessarily equate to infection, yet may be interpreted by providers as a significant finding (9, 10) and lead to unnecessary antimicrobial therapy and other interventions (11). Dialogue between the clinical laboratory and ID specialists is extremely helpful to gain a mutual understanding of the clinical actionability of test results, test cost and performance characteristics, and newly available tests for preemptive consideration.

## ROLE OF A DIAGNOSTIC STEWARDSHIP COMMITTEE

While not essential for performing DxS, a DxS committee can be helpful throughout all steps of stewardship as reviewed in this document. Such committees are an excellent mechanism to bring together stakeholders and build collaboration throughout the institution. A long-standing and common model for DxS stems from a laboratory utilization committee that encompasses a medical pathologist, lab staff administration, and physician/administrative champions (8, 12).

### Support

Implementation of DxS within healthcare facilities cannot succeed without institutional commitment and the support of stakeholders. Support includes time resources, financial resources (e.g., funding personnel time and software, interface, or new instrument purchases), opening provider communication channels, and championing stewardship activities (13). Partnering with a clinical champion can further aid in obtaining institutional buy-in and support.

### Stakeholders

Stakeholders in a DxS committee can include, but are not limited to, representatives of the major ordering provider groups (e.g., ED, hospitalists), ID physicians, AS colleagues, infection prevention and control experts, laboratory information systems (LIS) team, billing and compliance departments, and associated information technology teams. Specialty groups may not all be part of the core stewardship committee but should be invited to committee meetings when relevant tests are discussed. The potential for overutilization and overspending on molecular diagnostic tests, especially those without insurance reimbursement, can draw scrutiny from hospital administration, thus their inclusion in stewardship activities and support for the required resources are important.

If the laboratory has been approached by a particular subspecialty clinical group (e.g., intensive care unit providers, urologists) to request that a molecular microbiology test be made available, we recommend including that clinical team in the assessment process of the request. Furthermore, members of ID or AS teams can help evaluate the clinical utility of the test and possible risks for inappropriate antimicrobial usage if the new test is implemented. This could be done as a preliminary discussion before bringing the request to the full DxS committee or presented to the entire committee upfront. A robust review of developing a microbiology-specific DxS program specific to AR surveillance was created by the World Health Organization (WHO) in 2016 (14).

### Activities

Interdisciplinary collaborations are most beneficial for prioritizing tests to be stewarded, defining DxS initiatives (e.g., to decrease inappropriate test orders, optimize antimicrobial therapy, or reduce hospital-acquired infection rates, etc.), identifying specific populations that would most benefit from DxS, defining criteria for test ordering and approval, and determining how to implement stewardship policies (15).

DxS programs have been established at many institutions not only to help providers select the appropriate test for individual patients but also to assist in the management of patients based on test results. The WHO recognizes DxS as an integral part of AS programs and infection prevention and control activities in healthcare facilities (14). Suboptimal management of an infectious process increases antimicrobial resistance. Ultimately, optimal infection management requires an interdisciplinary approach and strong collaboration between the laboratory and AS team (16–19). Without the support of clinical teams, laboratories may not achieve the intended outcome of their testing initiatives (20, 21).

## UTILIZATION TRENDS THAT CAN BE DETECTED OR MONITORED

The following utilization trends are common testing issues that may be identified to initiate the stewardship process. Continued monitoring for such trends after implementing an intervention also enables performance and impact assessments (Fig. 1). Utilization trends can be assessed by annual volume comparison, finance reports with operating expenses and established budget comparisons, reimbursement denials reports, or turnaround time (TAT) trends.

### Overutilization

Overutilization is the utilization of testing services that exceed those reasonably expected to benefit the health of a patient, based on their clinical presentation and treatment needs. Overutilization can lead to delayed TAT, false positive results prompting inappropriate treatment (22), delayed diagnoses, unnecessary additional workup, and increased laboratory costs without providing the intended clinical utility. High volume tests are low-hanging fruit for evaluating utilization. Feedback from subspecialty providers, such as ID, may highlight negative clinical impacts due to test overutilization.

### Underutilization

Underutilization occurs when a test is ordered less frequently than would benefit patients, usually for a specific population or clinical presentation. Underutilization can lead to suboptimal, delayed, or erroneous diagnoses, or higher costs to obtain a correct diagnosis. Often, underutilization of a molecular microbiology test is accompanied by overutilization of another method to detect specific pathogens (e.g., viral culture being routinely ordered for Herpes Simplex Virus or respiratory virus detection instead of PCR).

### Misunderstanding test performance

Misunderstanding test performance often results in suboptimal ordering or suboptimal interpretation of test results, which can lead to inappropriate clinical management. For example, if a test is indicated in a specific patient population (e.g., *Aspergillus* PCR test in hemato-oncology patients), but is being ordered by general practitioners, misunderstanding test performance should be suspected.

### High-volume send-out tests

Monitoring send-out test volumes can help identify inappropriate utilization patterns which may merit further stewardship. When a high-volume send-out test is used appropriately or is essential to the diagnosis or management of a specific population cared for at the institution, this may prompt an evaluation for possible implementation of the test in-house. Send-out test monitoring and governance may be more accessible for tests that are built in the LIS for ordering and reporting, allowing them to be tracked using LIS data and analytic reports. Whereas, miscellaneous reference test ordering, which is often made available to providers as an option to accommodate rarely ordered or newer tests, may be more challenging to monitor. Newer molecular diagnostic tests often fall into this category and may present DxS opportunities. Reference laboratories can serve as a valuable resource in providing test volume and utilization reports to client laboratories.

### Specimen types, collection, and transport

A preferred specimen type for a given molecular test may depend on the clinical situation, including patient population, syndrome to be diagnosed, or feasibility of specimen collection. Order analysis, specimen rejection rates, and rates of using suboptimal specimen disclaimers can highlight trends in suboptimal specimen type selection. Additionally, the timing, collection method, container, and transport conditions should be considered. Suboptimal pre-analytic collection and processing steps can lead to inaccurate or delayed diagnoses, resource waste, or increased healthcare costs. For many tests, more than one specimen type is accepted. For example, vaginal swab specimens are recommended over urine for the diagnosis of *Chlamydia trachomatis* and *Neisseria gonorrhoeae* in anatomically female patients (23). However, testing urine is preferred over no test at all. At an institution where urine is routinely collected, the stewardship process can help determine if this practice is due to providers not knowing that vaginal swabs are more sensitive than urine vs strong patient preference that precludes the more invasive swab collection.

### Low rates of reimbursement

Tests with denials and low reimbursement rates may be prioritized for stewardship to help ensure the testing is clinically indicated. Inappropriate utilization may lead to reduced reimbursement and higher patient bills.

Reimbursement is complicated and may differ depending on the payor, state, and local regulations. Laboratory directors should be aware of their local Medicare Administrative Contractors (MAC) requirements (24). MACs are determined by geographical location and are set at the state level. Reimbursement between MACs varies. For example, some MACs do not reimburse large respiratory panels, urinary tract infection molecular panels, and other multiplex panels. Repeat testing ordered on the same patient on the same day may also not be reimbursed. Palmetto MolDx, the MAC for many states, requires additional documentation from laboratories demonstrating clinical validity and clinical utility (25). Additionally, laboratories should be aware of tests that do not have associated current procedural terminology (CPT) codes and whether such a test is likely to be reimbursed. Another consideration is the practice of prior authorization for laboratory testing. While already common for certain genetic tests, prior authorization is being applied by insurance providers to an increasing number of other high-cost molecular tests in an effort to manage healthcare costs. As the reimbursement landscape evolves, microbiology laboratories should be familiar with the concept of prior authorization, associated administrative challenges, and changes to the workflow of ordering and performing tests (8, 26).

## METHODS FOR IDENTIFYING HIGH-PRIORITY TESTS FOR STEWARDSHIP

Laboratories are tasked to make an array of molecular microbiology tests available to providers for routine and specialized testing. Consequently, institutional molecular microbiology test menus often evolve through the identification of new or improved diagnostic tests suitable for the care of the patient population served. Such tests may merit special attention for DxS as providers learn how to optimally integrate their use into clinical care, sometimes with limited clinical published data. However, DxS is equally important for existing tests.

Identifying high-priority tests for stewardship includes assessing reported clinical utility, clinical impact, and best practice utilization guidelines compared to those at the institution, overall test performance, laboratory feasibility, and costs. The laboratory should discuss these parameters with the appropriate clinical teams and understand when, how, and if a test can improve patient care for a particular population or clinical indication (1, 2, 27). In this section, we will list and describe the tools for identifying high-priority tests for DxS. Examples of the types of stewardship challenges that may be detected through the below processes are detailed in Table 1.

**TABLE 1:** Common stewardship challenges

Stewardship challenge	Example scenarios	Potential interventions
Overutilization	Ordering respiratory multiplex PCR testing on all admitted patients regardless of symptoms	Limit availability in order sets Restrict ordering to certain providers/locations Adopt an approval process
Underutilization	Failing to order HCV molecular testing to establish a diagnosis in HCV antibody-reactive individuals	Adopt an automatic reflex testing algorithm (with institutional approval) Adopt reporting language that facilitates follow-up testing
Target redundancy	Simultaneously ordering SARS-CoV-2, influenza, and/or RSV standalone RT-PCR testing along with respiratory multiplex testing	Implement cancellations based on previous test(s) ordered Curate order sets and test menus
Limited clinical impact	Ordering tests with lengthy turnaround times (i.e., 16S or 18S rRNA PCR for bacteria or fungi) in patients without planned follow-up	Limit availability in order sets Restrict ordering to certain providers/locations Adopt an approval process
Seasonal differences in utility	Ordering influenza testing in summer or arbovirus testing in winter	Limit availability in order sets Adopt seasonal approval processes Seasonally curate order sets
High cost/low reimbursement	Ordering broad-range pathogen panels in patients without clinical necessity	Restrict ordering to certain providers/locations Adopt an approval process Curate the laboratory formulary and order sets
Testing outside of established algorithms	Performing HIV qualitative molecular testing as a routine screen in lieu of CDC/APHL recommended serologic screening	Adopt reflexive algorithmic testing Restrict ordering outside of algorithmic testing Employ reporting with language that encourages appropriate algorithmic testing Use computerized clinical decision support to guide test selection
Little data on test performance characteristics	Mucorales PCR; specialty molecular panels offered by only a single laboratory	Gather input from DxS committee or key stakeholders Restrict ordering to certain providers/locations or clinical indications Request test validation data from vendor

### Stewardship of a new test: list of considerations

The decision to bring in or offer a new test is, itself, an act of DxS. We recommend that new test request assessments be considered a high priority for stewardship. There are several aspects that laboratories can assess to determine the appropriateness of offering a new test, including

- Clinical Utility and Test Requirements Assessment
  - Why is the test being requested?
  - What is the intended clinical application?
  - What is the anticipated clinical necessity and clinical impact?
  - Is the TAT sufficient for clinical impact?
    - Define TAT required to support the target clinical impact
    - Evaluate the appropriate location for testing to meet TAT needs (e.g., point of care, onsite hospital laboratory, central laboratory, send-out)
  - What is the reported test performance?
    - Ways to gather this information include speaking with the sales representative, reviewing package insert material, attending webinars and conference presentations on use of this assay at other institutions, performing a literature review, and discussing with other health systems that utilize this particular assay
  - Review laboratory resource and facilities feasibility (e.g., staffing, instrumentation, space, electric power needs, data management, TAT, electronic medical record (EMR) requirements) (28)
  - Review regulatory oversight, compliance, and quality management requirements for specimens coming from all locations under an institution’s Clinical Laboratory Improvement Amendments (CLIA) certificate for compliance and appropriateness (e.g., physicians’ offices, free-standing EDs).
  - Review if an ordering algorithm or other LIS support will be needed
  - Review the financial feasibility:
    - Obtain test volume estimates from anticipated users (e.g., a single provider, single department, hospital or system wide, inpatient or outpatient).
      - Consider how anticipated test volumes and reagent contracts may impact the cost-per-test
    - Perform cost analysis for the new test (29)
      - Laboratory costs include reagents, including calculations for anticipated waste due to reagent instability, particularly for low volume tests; instrumentation and maintenance contracts; personnel; overhead; quality control testing; proficiency testing requirements; implementation costs (verification or test development and validation, initial training and competency testing, order and test build, interfaces)
      - Review expected reimbursement
        - Does this test have an associated CPT code?
        - How and when can this test be billed?
        - Review test reimbursement from primary insurers to your system or any local rules (e.g., MAC restrictions)
      - Calculate the return on investment by accounting for laboratory costs (above), initial investment requirements and expected future cash flows (e.g., reimbursement prices and rates), and risks (e.g., anticipated testing volumes and the potential degree of inaccuracy of estimated volumes, potential denials rates) (30)

After deciding to offer a new test, a laboratory should collaboratively determine if the test will benefit from additional stewardship after go-live.

### Stewardship after test implementation (new or existing tests): list of considerations

In addition to the approaches listed above, there are several approaches that laboratories can use to identify, monitor, and prioritize newly implemented or existing tests that may benefit from DxS, including:

- Open peer-to-peer discussion with frequent users or the DxS committee (31)
  - Patient-facing providers are an excellent resource to identify tests whose use or interpretation requires further guidance and support
  - Case presentations that personalize and prompt investigation of broader utilization trends
- Review in house and send-out test utilization reports (see *Utilization trends that can be detected and monitored* section above for more details)
- Review appropriateness of predominant specimen types collected (see *Utilization trends that can be detected and monitored* section above for more details)
- Review provider questions/complaints for common or recurring themes
  - For examples, indications of a problematic diagnostic test or misunderstanding of the clinical utility of the test
- Review test reimbursement denials and duplicate testing trends
- Review trends in TAT delays
  - Is the TAT not set correctly? Is the TAT of the test meeting clinical needs?
- Review therapeutic management response to test results
  - Is the test prone to false-positive results that lead to over treatment? (32)
  - Do results routinely drive more targeted therapy or discontinuation of antimicrobial therapy versus overly broad or inappropriately narrow therapy?
  - Is the time to optimal antimicrobial therapy reduced?
- Review published literature and community resources to identify commonly misused molecular tests
  - Effective Test Utilization (ETU) (previously known as Choosing wisely) (33)
  - Guidelines from professional societies (e.g., guidelines that recommend specific testing, e.g., American Society for Microbiology [ASM] and Infectious Diseases Society of America) (34)
  - Literature of real-world examples
  - Online community resources such as professional society listservs, labtestingmatters.org (35)
  - Some reference laboratories provide useful online information on test requirements, interpretation, and utilization recommendations

## APPROACHES TO STEWARDSHIP

Once a priority test for DxS is identified, the laboratory can evaluate possible interventions. The best intervention depends on the clinical issues leading to the need for stewardship, setting and type of end users, type and quantity of resources available for supporting stewardship, and characteristics of the test. In this section, we review common DxS interventions that may be used independently or together.

### Education

The educational approach and information delivered should be customized to the institution and the role of each team member in the target audience for effective outcomes (34). For instance, optimal strategies for educating outpatient vs inpatient providers may differ. Often for outpatient services, there is a wide network of providers and clinics that require an education method that can be distributed broadly and where in-person education opportunities may not be feasible but may be considered when needed. Clinical providers on DxS committees are essential in distributing information and educating colleagues. Educational interventions may also be targeted toward the highest test users or departments via direct communication or laboratory in-services. This targeted approach can reduce molecular test overuse as has been demonstrated in *Clostridioides difficile* PCR testing (17, 36).

Educational efforts range from issuing mass communications such as laboratory bulletins, maintaining an informational test catalog, providing virtual or in-person didactic or in-service sessions to providers, embedding education within interpretative comments of test results, and implementing clinical decision support or electronic advisories at the point of test order (37, 38). Other resources for educating stakeholders include vendor-provided seminars, published society guidelines, and laboratory test directory for local or reference laboratories.

Education may also be targeted at improving the AS impact of molecular microbiology testing. Collaborations with AS provide opportunities to develop local treatment guidelines that incorporate the interpretation of organism detection and antimicrobial resistance (AMR) markers from molecular assays. Published guidelines are available as resources of expert information on results interpretation of AMR gene detection (39, 40). The partnership of laboratory and AS providers is a critical factor in achieving clinical impact with rapid assays for AMR detection (41).

### Laboratory formularies

One way of promoting DxS is to focus and limit which tests are available. This can be achieved by a “Laboratory Formulary” (8, 42–44). A Laboratory Formulary is a policy defining which tests are approved by the institution and in what circumstances they are approved. Similar to a pharmaceutical formulary which catalogs drugs that can be prescribed in each setting, it can be used to standardize and drive approaches to patient management through testing.

While akin to a “test menu,” Laboratory Formularies are more complex. They typically include tests performed both locally and those sent to reference laboratories. They may define the specific reference laboratory and clinical setting permitted for a send-out test. Decisions regarding whether a test is offered should consider clinical utility, TAT, availability of alternatives, and cost; tests may be stratified within the Laboratory Formulary based on these characteristics (44). Depending on the tier, different administrative controls may be put in place. For example, some tests may be accessible only to specific locations or providers, and some tests may require special authorization whenever ordered (8, 42). Tests deemed to have little clinical value can be omitted from the electronic ordering system. Laboratory Formularies should ensure that tests ordered as “miscellaneous send-out” tests are also evaluated for inclusion or exclusion.

Laboratory formularies are not static. As new tests are developed and testing needs evolve, formularies should be re-evaluated and edited. This requires regular review and having defined processes by which providers can submit proposed changes and provide feedback regarding gaps in the formulary. Given the complexity of clinical medicine, providers may need alternative ordering mechanisms to order off-formulary tests when clinically indicated (42).

### Test approval systems

Test approval systems are another form of administrative control that can drive appropriate utilization. Through this strategy, testing is either approved or denied based on pre-determined criteria. Generally, test approval systems can be classified as either manual or automated strategies, each with advantages and disadvantages.

Manual approval systems often involve review of the requested test by an expert or group of experts. This often includes designated individuals or a combination of medical directors of a laboratory, trainees in laboratory medicine, ID providers, and pharmacists. This approach has been shown to be effective for costly, nuanced tests such as cell-free metagenomic next-generation sequencing (45). An advantage of manual approval systems is that they allow reviewers to consider a patient’s entire clinical scenario and assess the test request in context with both clinical data and feedback from the requesting provider. A disadvantage is that this approach is labor intensive and can increase TAT. Manual approval processes may necessitate constant readiness of individuals who make approval decisions. This often means the process is managed by multiple individuals, which can potentially decrease the standardization of decision-making. Defined criteria requiring approval can improve the consistency of the approvers.

Automated approval systems via the EMR allow standardized and efficient approaches. These may be designed via electronic order entry question responses, automatic cancellations, or hard stops based on predetermined criteria (e.g., pre-approved clinical indication selection, canceling repeat testing within 24 h). Since criteria for these approval processes are more defined than “expert opinion,” they should be evidence-based and designed by expert users in collaboration with the laboratory at the institution. An example of one such approach is the utilization of cerebrospinal fluid cell counts to determine the acceptance of viral pathogen molecular panel requests (46, 47). Through a standardized interpretation of cerebrospinal fluid cell count parameters and the patient’s immune status, this approach has been shown to dramatically decrease low yield testing (47). Another broadly used approach includes electronic hard stops. For example, when employed for enteric bacterial molecular panels ordered within 3 days of admission, this approach has significantly decreased the number of unnecessary tests performed on inpatients (48).

A disadvantage of automated approval systems is a lack of flexibility and nuance. They may impart a “one size fits all” approach to DxS which may not always be appropriate. Attempts have been made to convert the appropriateness of more nuanced testing into something more standardized through scoring systems. For example, a scoring system to predict the appropriateness of bacterial broad-range 16S rRNA testing has been proposed (49). However, the rigidity of automated approval systems has led them to often be considered interventions of last resort.

### EMR ordering alerts and other health information technology tools

Computerized clinical decision support (CCDS) is a form of electronic intervention designed to alter the behavior of an ordering provider in the direction of a desired outcome. Examples of CCDS include electronic soft or hard stops, response or criterion-based test selection within a single order, electronic notifications, and redirections at the time of ordering. These notifications are often referred to as “best practice alerts” (BPAs) or advisories. To be most effective, notifications must target the right person with the right information at the right point in the workflow (50). For example, an alert that only fires when the laboratory receives the specimen for testing is suboptimal for targeting a specific order or collection action. Similarly, an alert only seen by a secondary group entering the orders on behalf of a provider (e.g., nursing staff) is unlikely to influence a change in the ordering decisions.

Effective notifications succinctly communicate why the intervention is occurring and facilitate a more appropriate course of action, which may be a different test or test cancellation. Notifications have been effectively employed to decrease inappropriate testing (e.g., *C. difficile* testing and syndromic molecular testing) and promoting appropriate testing (e.g., infectious hepatitis screening) (36, 51, 52). However, notifications that can be overridden (soft stops) often show only limited success (52, 53). As such, notifications are often coupled with one or more other DxS approaches. In the setting of inappropriate *C. difficile* molecular testing, notifications coupled with “hard stops” have shown great efficacy, particularly when compared directly to notifications without hard stops (36, 54). Notifications linked to a mandatory approval process (e.g., through pharmacy or laboratory medicine resident) have also shown success (54, 55). While very effective, there are often barriers to restrictive CCDS implementation. They require buy-in from hospital leadership and other relevant parties. The benefit must be greater than the potential negative impact of contributing to provider alert fatigue.

Aside from notifications, CCDS may provide ready information at the time of electronic ordering. In some health information systems, CCDS tools can link (e.g., with mouse click or hover over an icon) to order information or an institutional guideline. Specimen collection requirements with photographs of proper collection devices may also be embedded in EMR test catalogs and order entry modules for pre-analytical support. Such tools allow laboratories to target frequent issues in real-time at the order entry interface.

CCDS may also be implemented in the form of automated reflex testing algorithms. These algorithms may allow broader or more expensive tests to be ordered after more narrow or less expensive testing is non-diagnostic, or automatically trigger follow-up testing after initial testing meets criteria. The algorithmic approach has shown encouraging results not only in medication prescribing but also in laboratory test ordering (56).

### Reinforcing specimen requirements

Laboratories are responsible for educating ordering providers on the importance of specimen collection requirements for microbiologic analysis and for ensuring that the specimen has been appropriately selected, collected, and transported to the laboratory (CLIA standard §493.1240) (57). Ideally, education and communication should occur before the specimen is collected. This can be accomplished via a procedure catalog within the EMR or in other areas that collectors and providers electronically frequent, including the CCDS approaches described above.

Additionally, when a specimen is incorrectly collected or handled, the laboratory is responsible for upholding specimen requirements, including rejecting specimens and requiring recollection. For new molecular microbiology test offerings, provider education programs are essential to introduce end users to new diagnostic tests and can be used to suggest appropriate specimen collection strategies (58).

### Test result reporting and interpretation

Accurate result interpretation relies on the specialized knowledge of clinical indications for testing and assay performance characteristics, which must be conveyed to the providers who receive the results. Interpretative judgment and reporting strategies are particularly important in infectious disease testing and require oversight by an experienced clinical microbiologist, with input from ID or AS experts, to incorporate the interpretative guidance for the results into clinical care (34). When an organism with pathogenic potential is detected by a molecular microbiology test, it must be determined if the organism represents active infection, colonization, asymptomatic infection, reactivation in the setting of acute infection, chromosomal integration, prolonged shedding after infection, or other clinical scenarios (17). The following are methods to support accurate interpretation of molecular microbiology test results:

- The report should be clear and the significance of the results should be easy for providers to understand
- To aid correct interpretation, report comments may be employed (e.g., when *bla*_CTX-M_ gene results, the report should include the interpretation that an ESBL-producing organism was detected and that resistance to penicillins and cephalosporins is predicted) (39)
- Consider including a link in the report to further guide result utilization (e.g., linking institutional therapy recommendations when a report indicates a carbapenemase-producing organism was detected)
- Add report comments to alert providers to critically assess the result when an organism could be a false positive detection or analytically true positive detection but clinically insignificant (e.g., HHV-6 detected in CSF could indicate infection or non-infectious chromosomal integration in white blood cells depending on the clinical presentation and risk factors) (59)
- For broad-range molecular microbiology tests (e.g., metagenomic next-generation sequencing or 16S rRNA PCR with sequencing), add comments to guide cautious interpretation when nucleic acids are detected from common commensal or contaminating organisms. Comments encouraging correlating the molecular results with other clinical results may also help
- For some molecular test results, additional consultation may be necessary. For example, if there is a discrepancy between molecular and phenotypic tests (e.g., molecular test detects *S. aureus* and *mec*A gene, but antimicrobial susceptibility testing demonstrates oxacillin and cefoxitin susceptibility)
  - Discussion with providers often provides mutually beneficial information to arrive at an accurate determination of an organism’s clinical significance
  - Consultation may also support test interpretation of send-out testing for which the local laboratory has limited ability to alter the reports or add interpretative comments

## METHODS OF MONITORING PROGRESS, SUCCESS, AND UNINTENDED CONSEQUENCES

Like any other process in healthcare, molecular DxS interventions should be followed by a systematic, structured approach that provides feedback and monitors progress (58). Appropriate metrics are fundamental to guide and evaluate the effectiveness of the stewardship process. When selecting monitoring methods and metrics, the availability and capability of informatics resources must be considered. Prior to monitoring progress, the targeted outcome of the DxS project must be clearly defined, and the selected measures and specific performance metrics must accurately reflect the goals and interventions. Defining the methods of monitoring an intervention upfront ensures the intervention will be measurable and allows sufficient time for the development of supporting tools or reports. Table 2 shows examples of monitoring metrics that may be used to assess the progress or success of the molecular testing stewardship interventions.

**TABLE 2:** Case-based examples of metric monitoring after an intervention

Example scenario	Example intervention(s)	Metrics to monitor for impact and sustainability of the intervention(s)^*a*^
Overutilization: daily viral load monitoring on immunocompromised patients (e.g., CMV, EBV)	Targeted education regarding typical viral load dynamics and preferred frequency of testing Automated soft or hard stops for redundant orders within a set timeframe	Time between replicate orders by patient and service or provider Number of bypassed soft stops if applicable Provider feedback: monitor for signals of populations that merit more frequent testing, not originally considered, vs need for additional education
Underutilization: low volumes of testing for Parechovirus meningitis/encephalitis in young infants	Pop-up advisory offering Parechovirus PCR on CSF when Enterovirus PCR on CSF is ordered in patients under 3 months Clinical decision support order tool to guide selection of Enterovirus PCR and/or Parechovirus PCR on CSF based on patient age for the diagnosis of meningitis/encephalitis	Number of tests by type of test, order, and age Positivity rates and total positive diagnoses Monitor for unexpected trends toward increased testing and potential overutilization in older children Monitor seasonality and epidemiology of infections. Adjust or turn off the intervention, as appropriate.
Suboptimal testing: delayed orders for confirmatory testing by HIV-1 NAAT according to the CDC recommended algorithm	A reflexive order that automatically completes the CDC diagnostic algorithm and requires the appropriate specimen collection for all tests	Number of tests by type of test and order Number and percentage of return lab draw visits for HIV-1 qualitative NAAT Time from first test to completed diagnosis
Order issues: frequent calls to the laboratory for aid in finding the order for *Pneumocystis jirovecii* PCR	Modify the order synonyms in the EMR to include common terms (PJP, PCP, carinii) Modify the test name to include additional commonly recognized terms	Number and type of calls to the laboratory Number of other non-PCR *Pneumocystis* orders and miscellaneous orders placed instead of the intended order
Suboptimal specimen collection: increased detection of *C. difficile* colonization due to testing of semi-formed stool	Electronic collection instructions specifying required stool consistency Laboratory rejection of semi-formed stool according to a defined scale	*C. difficile* positivity rates Total *C. difficile* test volumes Specimen rejection or redraw rates Unfilled order rates due to no specimen being collected Provider reports of delays in *C. difficile* infection diagnosis
Suboptimal treatment: overuse of antimicrobial treatment based on metagenomic next-generation sequencing (mNGS) result interpretation	Implementing a workflow for approving mNGS orders Active clinical consultation and follow-up of all mNGS results	Antimicrobial treatment regimen before and after mNGS results (with expert interpretation of appropriateness if possible) Number of AS interventions made after mNGS Patient outcome monitoring Percentage of positive, negative, or commensal organism results for specific patient populations and sources Update approval parameters on a scheduled basis based on most current literature and institution-specific results.

^*a*^ Metrics may be monitored at a frequency appropriate for the system and the stewardship project (e.g., weekly, monthly, quarterly). These metrics should be performed for the specific test or analyte targeted by the intervention. Metrics should include a comparison of before and after the intervention. It may also be helpful to compare testing patterns of areas not targeted by the intervention vs those targeted by it.

According to CLSI document GP49-ED1, ideal performance metrics should have the following attributes (13):

- Accurately capture all relevant clinical and operational effects of the test or initiative under evaluation such as the impacts of the intervention(s), patient outcomes, hospital statistics, and other potential secondary outcomes
- Accurately exclude or adjust for clinical or operational changes unrelated to the intervention(s)
- Achieve sufficient statistical power
- Be concise and easy to interpret

For example, instead of monitoring TAT to measure overutilization of a test, an ideal performance metric would include comparing test order volumes to a benchmark or to a calculated target volume for the institution.

Data monitoring may include the analysis of direct measures (e.g., the number of tests performed for a specific target or analyte), random sampling of a subset of results, or by interpretative measures (e.g., appropriateness of antibiotic selection based on test results). Data can also be stratified by user or population, such as inpatient vs outpatient or different hospital sites within a system. Pareto diagrams, histograms, and pie charts can be useful stratification tools for metric analyses (60).

When it is not possible for the laboratory to obtain the desired specific information, data may be obtained from surrogate measures. For example, to monitor inappropriate use of a mycobacterial PCR, the number of tests ordered without a concomitant AFB culture could be monitored. The use of surrogate measures should be practiced with caution as they may not capture the desired information or may add confounding factors. Optimizing clinical utility is a primary goal of stewardship interventions but is one of the more challenging goals to monitor using discrete metrics. Such metrics are usually selected based on an assessment of the overall testing problem leading to poor clinical utility (e.g., overutilization, underutilization, misunderstanding test performance). These measures could include the number of tests performed, analyte positivity rates, antimicrobial prescriptions after resulting, reimbursement denials rates, and others.

Another useful measure of test utility is rates of result follow-up or viewing and promptness of reviewing. In a review of two studies on general follow-up of test results, lack of follow-up ranged from 20% to 62% for tests on inpatients and from 1% to 75% for ED patients (61). Another study found that in cases of suboptimal spinal epidural abscess diagnoses, 16.7% of diagnostic errors were associated with missed or delayed test result follow-up (62). Low follow-up rates can suggest low test utility and may signal the need for additional interventions.

With special regard to antimicrobial use, a subset of the prospective audit and feedback metrics often measured by AS teams can also be leveraged to evaluate the impact of any DxS interventions related to optimizing the use or interpretation of molecular microbiology tests. Additionally, antibiograms can provide indirect measures of the impact of cumulative AS and DxS interventions. Antibiogram results can also highlight unintended consequences or suboptimal progress of such interventions. Limitations to antibiogram monitoring are that antimicrobial resistance trends may be difficult to detect over short time periods and are influenced by antimicrobial susceptibility testing breakpoint updates, statistical power, and other biologic and epidemiologic factors.

As experts in understanding test performance, institutional workflow, and normal testing patterns, clinical microbiologists should take an active role in metric monitoring and result analysis for DxS interventions, with the help of informatics experts. They can utilize flow charts and data analysis to present progress and next steps to key stakeholders, while providing knowledge of the testing details (60). The next steps will either include a standardized review system or, when an intervention has failed to meet the intended goal impact, re-evaluation of the intervention.

### Conclusion

Stewarding the use of infectious disease diagnostic tests has always been a role of clinical microbiologists and laboratorians. With the rapid development of new technologies, especially molecular diagnostics, the benefits of stewardship have gained more recognition and the need for interdisciplinary participation in stewardship efforts has grown. While DxS committees are a powerful method to bring stakeholders together and determine the best stewardship approaches for an institution or targeted patient population, they are not required to implement successful stewardship. The approaches and tools reviewed in this report aim to provide microbiologists a foundation for building a multidisciplinary network to support stewardship projects, selecting and prioritizing molecular tests for stewardship, identifying effective and feasible interventions within the resources available, and methods to monitor outcomes and identify further interventions.
